# Building an Operational Definition of Grounding

**DOI:** 10.1177/15248380251343189

**Published:** 2025-06-27

**Authors:** Joshua Hammond, Wilson J. Brown

**Affiliations:** 1 Pennsylvania State University, Erie, USA; 2 Case Western Reserve University, Cleveland, OH, USA

**Keywords:** depersonalization, derealization, grounding techniques, trauma

## Abstract

Disorders with dissociative features are characterized by disruption of both branches of the autonomic nervous system, which may result in intense episodes of emotional dysregulation that induce a dissociative response. In clinical settings, grounding techniques are frequently cited as a primary approach to attenuate dissociative episodes within treatment sessions. However, grounding techniques have not been subjected to efficacy studies due to a lack of consensus regarding a measurable operational definition. This critical review analyzed the literature on grounding techniques with the intent to develop an operational definition of the term. Literature searches within ProQuest and PubMed yielded 1,894 results; 19 sources were identified for review following removal of duplicates and textual analysis. Sources were sorted into three categories: grounding techniques defined (21.05%, *n* = 4), mentioned (63.16%, *n* = 12), and described (15.79%, *n* = 3). Analysis of sources informed the development of an operational definition of grounding techniques, with emphasis on facilitation of physiological equilibrium, as well as tentative treatment guidelines for the implementation of grounding techniques in therapeutic settings.

Amid a therapy session, a client can experience numerous possible reactions to potential adverse or painful stimuli encountered in self-exploration. Among the most severe client reactions exhibited in a session is dissociation, which refers to a wide constellation of manifestations including: the experience of overwhelming emotion (Foa et al., 1992), out of body experiences (Brown, 2002), anhedonia (Frewen & Lanius, 2006), emotional numbing (Frewen & Lanius, 2006), amnesia (Nijenhuis et al., 2010), and loss of orientation to the present moment and surroundings (de Tord & Bräuninger, 2015). Dissociation may occur for several reasons during a session, and numerous techniques are available to reorient a person back to the present. Grounding techniques have been widely used for this purpose in therapy for decades but are sparsely mentioned, other than in passing, within the extant scholarly literature (de Tord & Bräuninger, 2015; Pierce, 2014).

Grounding techniques are postulated to interrupt dissociation through reintroduction of external stimuli (Dietrich, 2000). Attention to physical surroundings broadens awareness and redirects attention away from intense internal experiences (e.g., flashbacks, emotions, physiological sensations; Dunphy et al., 2014). Grounding techniques further address dissociation through soothing communications that facilitate calm and establish a sense of safety. Individuals who tend to dissociate are often instructed in grounding techniques to assist in the development of self-control over dissociative episodes (Yehuda, 2005).

Yet, grounding techniques have yet to be subjected to formal empirical investigation; to date, no study has established efficacy in support of this commonly used intervention. A primary obstacle to the study of the efficacy of grounding techniques is a lack of consensus regarding an operational definition, which impedes uniform empirical investigation. This critical review analyzes the extant literature with the following aims: (a) assess the current empirical understanding of grounding techniques in application to dissociative episodes and (b) formulate a cohesive operational definition of grounding techniques for use in future studies.

## The Current Diagnostic Definition of Dissociation

The *Diagnostic and Statistical Manual of Mental Disorders, 5th Edition* describes dissociation as disruption of the normal process wherein new information is integrated into identity. Specific psychological processes disrupted by dissociation include the integration of memory, emotional experience, perception of the current moment, goal-directed action, and motor control in the presence of adverse stimuli. The experience of dissociation may cause significant distress and impairment of functioning in one or more areas of a client’s life (DSM-5; American Psychological Association [APA], 2013).

## Nonpathological Dissociation

Most of the literature on the topic of dissociation focuses on pathological forms, expanding our knowledge of disorders that clinicians may observe in practice (Butler, 2006). However, there is a small, yet expanding body of research that recognizes dissociation as an adaptive process. In fact, normative dissociation is a common experience. What distinguishes nonpathological dissociation from pathological variants is the lack of associated subjective dysfunction and distress. Nonpathological dissociation, which tends to be brief and adaptive, allows individuals to consolidate information into their sense of self, solve problems, and lose themselves in a task (Butler, 2006). Mild forms of nonpathological dissociation, many of which are commonly experienced by most individuals, can even enhance memory consolidation, problem solving, and creativity (Butler, 2006; Schauer & Elbert, 2010).

Examples of natural nonpathological dissociation include hypnagogia and hypnopompia, which refer to the transitions between a state of wakefulness to sleep and from sleep to wakefulness, respectively. In a hypnagogic state, it is common to experience vivid dreams and for memories to be consolidated (Glicksohn et al., 1991; Jones et al., 2010; Sherwood, 2002). Unlike in pathological forms of dissociation, episodic memories are consolidated and incorporated into identity (Jones et al., 2010). Daydreaming, an intermediary state between fully focused thought and sleep, is a normal activity of the brain characterized by off-task thoughts (Ferrante et al., 2020; Poerio et al., 2016). This experience encompasses a broad range of activities that include imaginary scenarios, future planning, and fantasizing. The ability to place oneself in an imaginary scenario may allow an individual to solve problems or anticipate potential problems, temporarily escape a stressful environment, or cognitively reset to manage the rest of the day (Butler, 2006).

## Pathological Dissociation

Dissociation is often encountered in the aftermath of trauma amid experiences of intense emotional or physiological dysregulation. Dissociative experiences are conceptually organized into the categories of derealization and depersonalization. Individuals experiencing dissociation may feel as though they are floating outside of their bodies and witnessing events happening to someone else, or as if they are watching a movie (Bailey & Brand, 2017). This would fall within the category of derealization, which is an “experience of reality or detachment from one’s surroundings” (APA, 2013, p. 291). In contrast, depersonalization, which may occur in the presence of reminders of a traumatic experience (i.e., reexperiencing), is characterized by detachment from oneself, both in emotional experience and identity (APA, 2013; Marshall et al., 2019). Dissociative experiences occur within multiple disorders, accompanied by a wide variety of physiological symptoms.

Specifically, post-traumatic stress disorder, panic disorder, dissociative disorders, and borderline personality disorder each include dissociation or an associated feature of their respective diagnostic criteria (APA, 2013). Among the most common physiological characteristics of these disorders are autonomic hyperarousal (Dodd et al., 2022; ISSTD, 2011; van Wyk et al., 2016) and hypoarousal (Fiskum, 2019; Hetzel-Riggin & Wilbur, 2010; Quillman, 2011; Reijman et al., 2016). Hyperarousal is characterized by an overactive amygdala, which overstimulates the fight–flight–freeze mechanism leading to emotional dysregulation, hyperfixation, and overidentification of threats which increase distress (van Wyk et al., 2016). Hypoarousal consists of physiological withdrawal from the senses, leading to emotional numbing and disconnection from the bodily experience of emotion (Foa & Rothbaum, 1992; Fiskum, 2019; Quillman, 2011; Rauch & Foa, 2006). The autonomic dysregulation caused by hyper and hypoarousal can affect heart rate, brainwave states, and the physiological perception of emotion (Bailey & Brand, 2017; Bangel et al., 2017; Fiskum, 2019; Quillman, 2011).

Dissociation interferes with the integration of experience into memory and identity (APA, 2013; Brown, 2002; Sierk et al., 2019; van der Hart & Horst, 1989). These memories are thus rendered inaccessible and could lead to maladaptive coping via avoidance when in the presence of stimuli related to the inaccessible memories (Brewin, 2001; Frewen & Lanius, 2006). According to cognitive (Brewin, 2001; Frewen & Lanius, 2006; Rauch & Foa, 2006), dual representation (Brewin et al., 1996; Brewin & Holmes, 2003), and emotional processing theories (Brewin & Holmes, 2003; Rauch & Foa, 2006), a pervasive sense of fear impedes the ability to process information and higher order executive functioning. Following a traumatic experience, behavioral and cognitive avoidance offer immediate, short-term relief from internal and external reminders of the trauma but compromise the natural emotional processing required for the healthy integration of the event into memory and identity (Brewin, 2001; Brewin et al., 1996; Frewen & Lanius, 2006; Rauch & Foa, 2006).

## Theories of Dissociation

### Traumatic Dissociation Theory

Traumatic dissociation theory was first articulated by Pierre Janet and holds that dissociation emerges from the inability to process vehement emotions, which results in a loss of orientation to reality and present awareness (Dell & O’Neil, 2015; van der Hart & Horst, 1989). The experience of overwhelming emotions leads to a constriction of attentional capacity, thus narrowing observation of psychological phenomena (Dell & O’Neil, 2015; van der Hart & Horst, 1989). Extreme emotions may result in the inability to access traumatic memories until exposure to a stimulus occurs that resembles the trauma (Dell & O’Neil, 2015).

### Structural Dissociation of the Personality Theory

The theory of structural dissociation of the personality is a continuation of traumatic dissociation theory (Moskowitz & van der Hart, 2020; Nijenhuis et al, 2010; Steele et al., 2005; van der Hart et al., 2005). This theory posits that there are innate, goal-oriented action systems that motivate specific behavioral responses (Nijenhuis et al., 2010; van der Hart et al., 2004, 2005). These “action” systems, which are ordinarily integrated and promote adaptive responses in the individual, become disrupted following trauma. The action systems then work to compartmentalize overwhelming emotions and memories of the trauma so the individual can continue to function (van der Hart et al., 2004).

Action systems have two distinct and dichotomous response pathways. The first pathway, known as the apparently normal part (ANP), supports the maintenance of daily functioning (Moskowitz & van der Hart, 2020; Nijenhuis et al, 2010; Steele et al., 2005; van der Hart et al., 2005). The second pathway, the emotional part (EP), maintains the fight or flight response and, in traumatized individuals, holds traumatic memories and associated emotions. Following traumatization, the EP may remain disconnected from the ANP to promote adaptive functioning, while spontaneously emerging when the individual encounters traumatic reminders (Nijenhuis et al, 2010; Steele et al., 2005; van der Hart et al., 2005). As a result of the splitting between these two parts, this theory proposes that individuals may dissociate and experience emotional numbing, amnesia, time loss, and loss of spatial awareness (Nijenhuis et al, 2010; van der Hart et al., 2005).

The theory of structural dissociation also conceptualizes dissociation on the spectrum of severity. The least severe form on this spectrum is called primary structural dissociation: “an alternation of a single dissociative part of the personality mediated by the action systems of daily life and a second (rather limited and rudimentary) part mediated by defense” (van der Hart et al., 2005, p. 416). Secondary structural dissociation is characterized by further division and fragmentation of the personality into one ANP and two or more EPs (Nijenhuis et al., 2010; van der Hart et al., 2005). The most severe form is tertiary structural dissociation, which is described as a continued fragmentation of the ANP and elaboration of the EP, which occurs when an individual’s cognitive and emotional capacities to integrate and process traumatic memories are insufficient to maintain a single ANP (Nijenhuis et al., 2010; van der Hart et al., 2005).

### Attachment Theory

Attachment theory posits that the attachment between an infant and primary caregiver profoundly impacts the capacities to form meaningful relationships and regulate emotions throughout life (Bayne & Thompson, 2018; Bowlby, 1977; Dell & O’Neil, 2015). Infants learn to modulate their emotions via coregulation with primary attachment figures (Bayne & Thompson, 2018; Dell & O’Neil, 2015). When an infant’s needs for emotional coregulation are not met, dissociation is an adaptive response to avoid the experience of persistent, overwhelming emotions (Dell & O’Neil, 2015). Notably, children who experience disrupted attachment are more vulnerable to dissociation later in life (Bayne & Thompson, 2018; Dell & O’Neil, 2015; Lyons-Ruth et al., 2006).

### Somatic Dissociation Theory

Traumatic dissociation and attachment theories offer explanations for how traumatic events and attachment disruptions increase the susceptibility to dissociation later in life. However, when it comes to addressing the bodily response to trauma, the theory of somatic dissociation proposes that disruptions of normal somatic functions manifest from stress-related disruptions of integrative consciousness (Bob et al., 2013). In other words, trauma can cause intense psychological and physical pain, as well as emotional dysregulation and other dysfunctions of the autonomic nervous system. Subsequently, dissociation acts as a defense mechanism to temporarily escape the physical, bodily effects of trauma through emotional numbing, lack of conscious awareness of trauma, seizures, and/or alterations in perception (Bob et al., 2013; Nijenhuis, 2004; van der Hart et al., 2005).

This theory is consistent with animal models of disorders with dissociative characteristics. Dissociation is a feature of multiple disorders, many of which are characterized by hyperarousal. When an animal receives an unexpected shock amid high levels of hyperarousal, it generates more fear than when the shock is expected (Foa et al., 1992). This leads to a greater sense of generalized fear and withdrawal to avoid exposure to the threatening stimulus (Foa et al., 1992; Luchetti et al., 2021; Moreira et al., 2013). Fear and other emotions evoke physiological responses in the body that can be overwhelming, leading an organism to instinctively numb their emotions in order to escape from the bodily reaction to the stimulus (Feeny et al., 2000).

During dissociative events, it is not uncommon to lose awareness of the present moment and orientation to reality (Silveira, et al., 2020). Once awareness of the present returns, the realization of lost time, as well as the lack of memory for events during the episode, can be quite disconcerting (ISSTD, 2011). When an individual experiences severe emotional dysregulation or dissociation during a therapy session, immediate intervention is required to calm and reorient them to the present. Once the individual de-escalates, it may not be appropriate to continue the session depending on the circumstances of the dysregulated or dissociative experience. Therapeutic grounding assists clients with recognizing the cycle between emotion dysregulation and dissociation, reestablishing awareness of the present and emotional control, and establishing stability during and between sessions (Brand, 2001; Brand et al., 2012; ISSTD, 2011).

## Therapeutic Grounding

Grounding techniques are a widely accepted, broad constellation of therapeutic tools used to help calm individuals and reorient them to the present. Asking questions to reorient an individual to the present or directing them to touch a sensory object amid a panic attack are common examples of these techniques. The purpose is to help individuals self-soothe and begin the process of regulating their emotions (Brand, 2001; Brand et al., 2012; de Tord & Bräunginger, 2015; ISSTD, 2011; Pierce, 2014). Essentially, these techniques calm and distract an individual from the experience of emotion dysregulation and subsequent dissociation. In the treatment of dissociative disorders, grounding techniques are often used in the early stages of therapy to help stabilize clients who dissociate; the use of grounding attenuates as the client improves in their self-regulation (Brand et al., 2012).

While widely used and referenced, “therapeutic grounding” remains an amorphous and elusive construct. For instance, grounding techniques are mentioned nine times in the International Society for the Study of Trauma and Dissociation’s (ISSTD) guidelines for treating dissociation and once within the American Psychological Association’s guidelines for treating PTSD (APA, 2017a; ISSTD, 2011). Neither organization defines or operationalizes grounding, and no guidelines for implementation are offered. It is also unclear whether these guidelines refer to sensory or cognitive grounding techniques. In the clinical practice guidelines for PTSD published by the Veterans Affairs Administration (U. S. Department of Veterans Affairs, 2023) and International Society for the Study of Traumatic Stress (ISTSS, 2019), grounding techniques are not mentioned at all.

The principal reason for vague conceptualization and presentation of grounding techniques is the lack of a cohesive definition that permits measurement. Alexander Lowen introduced the first definition of grounding as “being based on the interaction of the mind and body . . . human beings are physically, emotionally, and energetically grounded to the earth . . . all energy finds its way into the earth this is the principal of grounding . . . a grounded person has body awareness . . . has feet on the ground . . . and is in touch with reality” (de Tord & Bräuninger, 2015, p. 16). Grounding is based on the principle that the body is the person, and treatment must involve using the physiology of the body to help regulate internal processes. As such, grounding techniques, which rely on simple sensory input, facilitate the regulation of internal processes to maintain orientation to the present amid the susceptibility to experience dissociation within associated psychological conditions (Pierce, 2014). Another definition proposes that grounding techniques use basic bodily movements and cognitive processes, such as questioning and information retrieval, to integrate bodily and emotional experiences, which creates a sense of well-being, stability, and orientation to the present at physical, emotional, cognitive, sensory, and social levels (de Tord & Bräuninger, 2015). This definition of grounding techniques focuses on the integration of various senses to be fully present.

Both definitions appear difficult to operationalize for evaluation. Lowen’s (de Tord & Bräuninger, 2015) definition emphasizes connectedness to the ground and discharging energy into the ground to feel more connected to the present moment. A major limitation of this definition is the rather vague conceptualization of energy; more specifically, how much energy needs to be discharged to establish present awareness? The concept of energy, the process by which it is discharged, and connectedness to the ground require operationalization to facilitate meaningful observation. Similarly, the integration of bodily and emotional experiences is a key to the latter definition, but the term integration is not defined and thus cannot be measured. What constitutes a “sense of stability” within this definition is also ill-defined. Without this clarity, there are obstacles to gathering uniform empirical data to establish the validity of the definition. Another common description of grounding is an intervention that reorients the person to the “here and now” (Pierce, 2014). This description of grounding techniques, at first glance, appears technically congruent with that of mindfulness.

While most of the literature discusses grounding techniques as basic sensory input, cognitive grounding techniques are also mentioned (Smout, 2018; Welfare-Wilson et al., 2021). These techniques often use simple prompts to promote cognitive recall, provide information to reorient the individual to the present, and focus their attention on an object, such as the chair they are sitting in, to bring sensory and cognitive elements together (Farrell & Taylor, 2017; Paulik et al., 2020; Smout, 2018; Welfare-Wilson et al., 2021). The nature of these techniques is similar to the non-elaborative self-awareness observed in mindfulness techniques. However, cognitive grounding techniques are scarcely described in the literature, unlike mindfulness. The resources an individual requires as a precondition for cognitive grounding techniques to be effective may be rendered inaccessible during a dissociative episode (Baumard et al., 2025).

Mindfulness is a well-defined and studied set of techniques that focuses on bringing an individual to a state of non-elaborative self-awareness and relating it to the individual’s own experience (Ottoboni, 2013). The process of non-elaborative self-awareness is focused on a set of metacognitions about how an individual is connected to others and themselves (Siegel, 2010). This emphasizes complex cognitive processes, including memory recall which may be rendered inaccessible during a dissociative episode (Wang et al., 2019; Zerubavel & Messman-Moore, 2013). Not surprisingly, grounding techniques are mentioned in mindfulness literature with little emphasis or description (Zerubavel & Messman-Moore, 2013). Yet, grounding techniques may be distinct from mindfulness, as the former operates via basic sensory input (de Tord & Braüninger, 2015), which remains accessible during dissociation. However, many mindfulness concepts and strategies are operationalized, whereas grounding techniques are not. Indeed, grounding strategies, techniques, and exercises are all used interchangeably in the literature to refer to the same thing (Smout, 2018; Zerubavel & Messman-Moore, 2013).

Notably, because grounding techniques lack an operational definition that would permit evaluation of efficacy and effectiveness, evidence for utilization is largely anecdotal from a clinician or client perspective. As such, grounding cannot be empirically evaluated and subjected to the same level of scrutiny required of evidence-based interventions. Similarly, the clinical application of grounding is not sufficiently outlined. Grounding techniques are largely mentioned in passing in the extant literature, which presents challenges to intervention researchers and clinicians who work with populations prone to dissociation alike. The focus of this critical review includes a discussion of therapeutic grounding in the extant literature and a synthesis of an empirically measurable operational definition of grounding techniques for use in future studies.

## Methods

This critical review was conducted in two databases, PubMed and ProQuest. The results included books, dissertations and theses, and peer-reviewed journal articles. Peer-reviewed publications were included in the current review if their abstract contained the relevant search terms. Peer-reviewed publications that contained relevant search terms were excluded from the review if the use of grounding techniques did not apply to therapeutic applications. All search results were cross-referenced to eliminate duplicates.

PubMed and ProQuest were chosen because of advanced search and database selection functionality, which increased the likelihood of identifying relevant results. Grounding techniques, exercises, and strategies are terms that are not unique to psychology, social work, counseling, and psychiatry; the ability to focus results within certain fields increased the likelihood of finding relevant sources for evaluation.

Google Scholar was excluded for consideration as a database for this study because the parameters used for the PubMed and ProQuest searches could not be applied to this database. Google Scholar does not have the ability in its advanced search feature to narrow the search terms to those appearing in the abstract. While this database would provide a wide range of information from numerous disciplines, the lack of ability to string Boolean search terms together and limit the search to the abstract would not allow the results garnered from this database to abide by the same parameters as the other databases. The initial search was conducted on July 16, 2023 with an updated search conducted on November 26, 2024.

Search terms focused specifically on the research of grounding for disorders with dissociative characteristics. Search terms included the following: grounding techniques OR grounding strategies OR grounding exercises AND dissociation, grounding techniques OR grounding strategies OR grounding exercises AND depersonalization, grounding techniques OR grounding strategies OR grounding exercises AND derealization, grounding techniques OR grounding strategies OR grounding exercises AND emotion dysregulation, grounding skills AND dissociation, grounding skills AND depersonalization, grounding skills AND derealization, grounding skills AND emotion dysregulation. After the removal of duplicate results, all sources were subjected to a textual evaluation to determine whether they discuss grounding within a therapeutic context. Sources were removed if they did not refer to grounding in a therapeutic context. All sources must be in English.

Sources that met the inclusion criteria were sorted into three general categories: defining therapeutic grounding techniques, describing therapeutic grounding techniques, and mentioning therapeutic grounding techniques. The final sources were compared across multiple criteria: general features of the source (e.g., type, research design, pertinent results), context in which therapeutic grounding is referenced, theme of reference, and relevance to the extant literature.

## Results

Initial database searches yielded a total of 1,894 potential sources. When the search results were cross-referenced to eliminate duplicates, 1,568 sources were eliminated, leaving 326 sources. The remaining sources were analyzed to determine if they met the inclusion criteria; 307 were eliminated leaving 19 sources for further analysis. The final 18 sources are comprised of 14 peer-reviewed journal articles, 4 book chapters, and 1 dissertation. Of these sources, 4 define grounding techniques (Table 1), 12 mention grounding techniques in passing (Table 2), and 3 describe grounding techniques (Table 3).

**Table 1.** Sources that Define Grounding Techniques.

Authors	Design	Description	Theme
de Tord and Bräuninger (2015)	Theoretical exploration of the use of grounding techniques in dance movement therapy	The original definition of grounding techniques refers to the connection of the person to the ground, which orients them to reality. This definition, like others, does not provide a mechanism for empirical observation.	Restoring the relationship of the body to the ground in order to increase present moment awareness.
Miller-Karas (2023)	Book chapter	Grounding is defined as the relationship between an individual’s body and present moment awareness. This can be achieved through sensory input through part of or the whole body in relation to *s* surface.	Restoring the relationship of the body to a surface in order to increase present moment awareness.
Ramberg and Wrangsjö (2002)	Book chapter	Grounding techniques support inner psychological functioning by connecting the individual to the relationship of the body to the ground.	Restoring the relationship of the body to the ground.
Welfare-Wilson et al. (2021)	Literature review	Grounding techniques utilize the five senses and can be used to mitigate aversive reactions to restore an individual to the zone of optimal functioning.	Restoring physiological equilibrium.

**Table 2.** Sources that Briefly Mention Grounding Techniques.

Authors	Design	Description	Theme
Bayne and Thompson (2018)	Theoretical development of counseling approach	It takes time to determine what grounding techniques are effective for the client.	Determining grounding technique’s usefulness for client.
Brand et al. (2012)	Survey of clinicians	Grounding techniques are used to help stabilize dissociative individuals.	Usage in therapy.
Burrows (2013)	Case study	Grounding techniques help trauma survivors develop a strong foundation for treatment.	Usage in therapy.
Dietrich (2000)	Literature review	Grounding techniques may help stop intrusive thoughts.	Usage in therapy.
Dunphy et al. (2014)	Qualitative analysis of participants’ experiences	These techniques can be used to promote body awareness.	Usage in therapy.
Kaur et al. (2016)	Case studies	Grounding techniques are useful for individuals who struggle with cognitive techniques. Grounding techniques are used interchangeably with several other terms.	For usage in therapy.
Givens (2015)	Phenomenological Interviews	Grounding techniques can help an individual attune to themselves.	Usage in therapy.
Payne et al. (2016)	Literature review of techniques used in dance/movement therapy	Grounding exercises can help develop a sense of self and body awareness.	Used as one set of techniques in an array of active methods to promote body awareness.
Standish (2015)	Case study	Grounding techniques can be used to reduce severe anxiety.	Usage in therapy.
Stirling and Andrews (2021)	Case study	Grounding techniques can increase body awareness	Usage in therapy.
van Minnen et al. (2014)	Theoretical development	Grounding techniques can reduce distress and allow treatment to resume.	Usage in therapy.
Yehuda (2005)	Case study	Grounding techniques are the foundation of identifying dissociation.	Psychoeducation.

**Table 3.** Sources that Describe Grounding Techniques.

Authors	Design	Description	Theme
Farrell and Taylor (2017)	Literature review	Grounding techniques could be used to mitigate adverse reactions when learning about trauma. Some grounding techniques may include running cold water over your hands or taking a sip of water.	Grounding techniques can be useful when teaching about trauma.
Paulik et al. (2020)	Case series	Grounding techniques provide a way to stop dissociation. It can involve interacting with a number of sensory stimuli (touching an object, essential oils, a squeezy ball, etc.)	Interaction with different sensory elements.
Smout (2018)	Book chapter—case study	Grounding strategies involve cognitive recall and basic sensory input. Such as naming things you can see, smell, touch, taste, and hear.	Cognitive recall of sensory information.

## Discussion

Grounding techniques are integral to clinical interventions for individuals who have disorders with dissociative characteristics, but no empirical data exist for their use (de Tord & Bräuninger, 2015). This stems from the lack of a concrete operational definition that permits uniform evaluation. The lack of concerted empirical support for therapeutic grounding limits the utility and applicability of associated techniques, despite wide anecdotal use and success. The present review confirms the paucity of useful literature on grounding techniques with identification of 18 sources that met inclusion criteria. Notably, 12 of these sources only briefly mention therapeutic grounding, and the remaining six sources vary widely in presented definitions and descriptions of grounding techniques. Three conclusions can be reached from the characteristics within descriptions of grounding from included sources to inform a more cohesive, succinct definition of therapeutic grounding.

### Finding 1: Abstract Definitions and Lack of Centralized Terminology

A major issue in synthesizing the extant research on grounding techniques is that there is no centralized terminology. While grounding techniques, strategies, and exercises appear interchangeably in the literature, these are not the only terms that are used. One source further referred to elements, activities, and methods within the discussion of therapeutic grounding (Kaur et al., 2016). This lack of consistency significantly obfuscates uniform definition and empirical investigation of therapeutic grounding for dissociation. One recommendation for the field is to centralize the terminology for grounding techniques, which would allow research on these techniques to mature and be more accessible.

One of the observed definitions of grounding techniques appears to be more amenable to empirical investigation than others, with emphasis on the utilization of the five senses to mitigate aversive reactions to trauma-related stimuli and return the individual to the zone of optimal functioning (Welfare-Wilson et al., 2021). Specifically, the concept of the zone of optimal functioning is most compatible with empirical investigation. Also known as the zone of optimal arousal (Siegel, 2010), this concept postulates that physiological equilibrium permits maximum self-control. Hyperarousal and hypoarousal are physiological deviations from the zone of optimal functioning, which result in behavioral excesses and deficits. Ecological assessment of physiological states in real time provides a proximal method to observe the return to the zone of optimal functioning (Back et al., 2022). One conceptual limitation is that much of the literature on dissociation focuses on hyperarousal, leading to a distinct lack of clarity on the measurement of effective interventions for hypoarousal. It may be possible to measure the effectiveness of a grounding technique on hypoaroused individuals through the use of vagal tone. This would permit future researchers to compare the effectiveness of grounding techniques for dissociative episodes characterized by both hypo and hyperarousal. It is necessary to understand the physiological processes by which grounding techniques may be measured and how they relate to the zone of optimal functioning. By learning how these processes work, it is possible to understand how basic sensory input can be utilized to return an individual to the zone of optimal functioning.

In the parlance of the Community Resiliency Model (CRM) and Trauma Resiliency Model (TRM), grounding techniques are referred to as grounding skills. Their definition of grounding is similar to Lowen’s, with the primary difference being that these models emphasize sensory input through part of or the entire body and the individual’s relationship to a surface rather than the ground (Miller-Karas, 2023). The focus of these models is on improving resilience in individuals and communities outside of the context of a therapy session. When considering this definition, there are two caveats that apply.

Firstly, the conceptualization of grounding within CRM and TRM is as a skill that targets the effects of trauma in individuals and communities by stabilizing the nervous system and enhancing resilience (Miller-Karas, 2023). While grounding is referenced as a potential intervention for individuals who dissociate, it is more often described as a daily self-regulation strategy than as a therapeutic technique utilized by practitioners to interrupt severe dissociative episodes (Miller-Karas, 2023). Our review focuses on grounding techniques administered by practitioners in such cases, rather than grounding as a skill to be learned by the client for daily practice and use.

Secondly, the conceptualization of grounding as a skill to be learned in CRM and TRM is atheoretical and similarly challenging to investigate. Efforts to dismantle these models and assess the effectiveness of their therapeutic strategies, including grounding, are scarce. Thus, research investigating the effectiveness of grounding within this framework is also scarce. If such research were available, extrapolation to dissociation in individual therapy contexts would offer limited information.

### Finding 2: Therapeutic Grounding Includes Sensory Input

A common feature that emerged among identified definitions of therapeutic grounding is that associated techniques utilize basic sensory input and prompts for simple cognitive recall (Farrell & Taylor, 2017; Paulik et al., 2020; Smout, 2018; Welfare-Wilson et al., 2021). Engagement with external stimuli appears to shift focus away from distressing internal stimuli that initiate and maintain dissociative responses, consistent with extant theories on the matter. Some sources hold that grounding techniques can be used to stop intrusive thoughts and emotions (Dietrich, 2000; Standish, 2015; van Minnen et al., 2014). In the few instances where a specific type of grounding technique is briefly mentioned in the literature, it is done so through the lens of sensory input. One technique that is mentioned as being effective is asking an individual to describe the room (Smout, 2018). Responding to this query engages multiple sensory modalities, including the auditory, visual, tactile, olfactory, and kinesthetic senses, in addition to the salient cognitive processes for encoding, retrieving, and generating information. Grounding techniques draw the attention of the individual outward through engagement with available sensory stimuli to encourage reorientation to the present. Directing attention outward leads to a natural distancing from emotions and intense physiological sensations—a valuable capacity for healthy emotion regulation. Grounding techniques may also facilitate self-control and safety if such techniques are independently applied successfully.

### Finding 3: Hyperarousal, Hypoarousal, and Emotion Dysregulation

Extant literature relevant to therapeutic grounding clearly indicates that disorders with dissociative characteristics share features of autonomic hyperarousal and hypoarousal. These changes are persistent and exist even when individuals are not actively under threat of violence. Individuals with these disorders experience low heart rate variability and high alpha–theta ratios (Bangel et al., 2017; van Wyk et al., 2016; Wahbeh & Oken, 2012). Individuals with these disorders are also more likely to experience the somatization of emotion, which can manifest itself through increased respiration, sweating, an inability to sit still, and insomnia (APA, 2013; Bailey & Brand, 2017; Becker & Zayfort, 2001). There are many physiological alterations in arousal patterns that are symptoms of these disorders—all of which represent targets for measuring the effectiveness of grounding techniques.

Emotional dysregulation frequently appears as a feature of dissociation. This is evident in the duality of hyper and hypoarousal, which are interrelated phenomena (Frewen & Lanius, 2006). The experience of hyperarousal can trigger hypoarousal, which may indicate that emotional numbing and other characteristics of hypoarousal may occur at elevated levels of arousal when the body tries to reestablish equilibrium (Frewen & Lanius, 2006). Hyper and hypoarousal are tied together by the fight–flight–freeze mechanism, and in the latter state, the individual may withdraw their awareness of their current environment in order to escape the presence of overwhelming stimuli (Brantbjerg, 2012, 2021; Frewen & Lanius, 2006). In either state of arousal, the cognitive resources of the individual are limited, while sensory capabilities remain intact (Yehuda, 2005). In order for cognitive processes and resources to function, the ability of the individual to use their senses must be established first (Baumard et al., 2025). One source reports that the grounding techniques integrated into the use of dance/movement therapy seemed to improve hypoaroused individual’s awareness of their current environment and sensory capabilities (Pierce, 2014). This seems to indicate that sensory information is essential for cognitive awareness.

The purpose of emotions is to motivate an individual to get to a place of physiological equilibrium and safety (van der Kolk, 2015). In disorders with dissociative characteristics, an individual disconnects from the somatization of emotion and cannot reach that state of equilibrium because their arousal pattern has been altered. Grounding techniques have been mentioned as a way to help calm people down (Givens, 2015; Yehuda, 2005). According to Bessel van der Kolk, “you can tell equilibrium has been restored when the physiology calms down” (van der Kolk, 2015, p.117). The need to reach a physiological state of calm is clear, as this provides a sense of rest and relief for the body.

Conceptualizing hyperarousal and hypoarousal as states of disequilibrium provides multiple ways to measure the effects of grounding techniques. Physiological changes resulting from dissociation are persistent (Frewen & Lanius, 2006; Foa et al., 1992). For example, therapeutic grounding may facilitate observable changes in brainwave patterns. It is acknowledged that individuals with dissociative characteristics have high Alpha–Theta ratios (Wahbeh & Oken, 2012). Theta waves are associated with emotional inhibition and Alpha waves with relaxation; in disorders with dissociative characteristics, there is an increased presence of Alpha waves and diminished presence of Theta waves (van Wyk et al., 2016). Grounding techniques may increase the presence of Theta waves relative to Alpha waves, thus restoring balance. The effectiveness of therapeutic grounding may also be indicated by an increase in the interbeat interval in the assessment of heart rate variability. A low interbeat interval indicates the presence of danger and that the body is mobilizing to provide enough energy to avoid harm (Wahbeh & Oken, 2012).

Hyperarousal and hypoarousal may present through a variety of physiological responses and psychological reactions (Bob et al., 2013; Foa et al., 1992; Nijenhuis, 2004; van der Hart et al., 2005), which necessitates consideration of the return to physiological equilibrium for inclusion within an operational definition of grounding techniques. Many of the reactions that are observed in individuals with these disorders occur after they are exposed to stimuli resembling their trauma. While certain stimuli can trigger a dissociative reaction, the introduction of neutral stimuli can enable the individual to calm themselves (Dietrich, 2000; Dunphy et al., 2014).

In summary, grounding techniques are generally used to help individuals establish safety, to recognize dissociation, and to help them calm down (Brand et al., 2012; van Minnen et al., 2014; Yehuda, 2005). According to the traumatic dissociation theory, when an individual dissociates, they experience a narrowing of their attentional field and focus inward (Dell & O’Neil, 2015). Somatic dissociation theory posits that dissociation is an adaptive response to escape the effects of trauma, including intense emotional dysregulation and physical distress (Nijenhuis, 2004). Both theories require consideration to understand the process of dissociation. In response to trauma, an individual hyperfocuses on their internal reality, including the psychological and bodily experience of emotion and distress. When this internal reality becomes intolerable and available coping strategies are untenable, dissociation occurs as a last resort to escape overwhelming internal stimuli. Yet, reorientation requires basic engagement of cognition as well, necessitating the consideration of both basic sensory and cognitive input in constructing a cohesive definition.

### Rationale for the Definition

Common descriptions of grounding techniques emphasize the use of cognitive recall and basic sensory input (Smout, 2018). During a dissociative episode, the process of memory consolidation is interrupted (Brewin & Holmes, 2003). As a result of the interruption of the memory consolidation process, access to routine cognitive processes such as recall and emotion regulation strategies is incapacitated by overwhelming emotions (Brewin, 2001; Frewen & Lanius, 2006; Nijenhuis & van der Hart, 2011; Zerubavel & Messman-Moore, 2013). However, the capacity for processing sensory information typically remains intact (Yehuda, 2005). For this reason, as well as the extant literature in support of the engagement with basic sensation to mitigate dissociation and reestablish normative arousal, the definition of grounding techniques predominantly emphasizes intervention via sensory modalities. In order for cognitive abilities to function, sensory integrity must be intact; otherwise, cognitive resources will remain inaccessible (Baumard et al., 2025). Thus, cognitive grounding and mindfulness techniques are not considered in this definition. Convergently, present moment orientation and awareness of surroundings are also not considered due to intact cognitive processes as a precondition for both. Cognitive grounding and mindfulness techniques may be appropriate for reality testing after physiological equilibrium has been established. It remains possible that cognitively focused grounding techniques may prove useful when cognition and memory remain accessible. The following definition presents a foundation for the empirical investigation of grounding techniques by incorporating two critical concepts: (a) sensory processing remains intact in individuals who dissociate and (b) represents the most direct strategy to reestablish physiological equilibrium.

### Proposed Definition

*Therapeutic grounding*: A constellation of techniques that engage one or more of the five basic human senses to return an individual to a state of physiological equilibrium.

### Implications for Future Research

Currently, there is no available systematic research on the effectiveness of grounding techniques. The current, predominantly anecdotal consensus is that such techniques work, but the putative mechanisms of effectiveness have yet to be elucidated. The proposed definition anchors the assessment of grounding techniques to observable physiological changes, which permits applied investigation of grounding techniques in ecologically valid contexts. Indeed, assessment of physiological biomarkers in session is already enhancing outcomes of standard evidence-based practices (Back et al., 2022). It is important to note that these assumptions have not been empirically evaluated to determine their validity and require additional scrutiny to determine the extent of their applicability.

In the extant literature, there is no distinction between grounding techniques for hypoarousal and hyperarousal. The lack of differentiation among techniques for differing levels of arousal is an area for future research. One factor that could complicate differentiating between techniques for hypo and hyperarousal is that hyperarousal can trigger hypoarousal (Frewen & Lanius, 2006). Much of the literature focuses on hyperarousal, which has adversely impacted our understanding of hypoarousal and interventions for individuals experiencing hypoaroused dissociation.

One of the difficulties in conducting this review was identifying relevant sources, because there is no centralized terminology to refer to grounding techniques. It is possible that the proposed definition could facilitate a centralization of terminology. At minimum, the proposed definition is a starting point for a uniform nomenclature of grounding that can be refined through future study. Across the reviewed sources, the terms therapeutic grounding and grounding techniques appeared to be most widely used and accepted. For uniformity, it is recommended that these terms be used exclusively, relative to other terms, in future discussions of grounding techniques.

It is possible that alternative definitions of grounding could be studied with self-report measures. However, a specific self-report measure for assessing deviation from and return to psychological equilibrium does not exist. While existing measures, such as the difficulties in emotion regulation scale (DERS), predominantly assess dimensions of experiencing and responding to emotional dysregulation (Gratz & Roemer, 2004), the development of instruments that specifically operationalize and target dimensions of grounding compatible with self-report methodology (e.g., connectedness, energy discharge) is needed.

The conceptualization of grounding presented above aligns with contemporary research on targeted physiological interventions. Indeed, multiple studies monitor physiological markers of hyperarousal as a metric of intervention effectiveness *in vivo*, and targeted physiological interventions for these disorders generally demonstrate significant reductions in reactivity (Back et al., 2022; Dawson et al., 2014; Docteur et al., 2021). Understanding hyperarousal as a state of persistent disequilibrium thus advances knowledge of the physiological aspects of dissociation and the specific grounding techniques that address each biomarker.

### Clinical Implications

To date, there are no existing clinical guidelines for the usage of grounding techniques in therapy. As such, a tentative set of guidelines for the use of grounding techniques with individuals who have disorders with dissociative characteristics was developed from the analysis of the therapeutic applications of such techniques within the reviewed literature. These guidelines are presented below:

(1) Provide psychoeducation to recognize triggers and signs of dissociation and emotional dysregulation, and introduce grounding techniques as a coping strategy to produce a sense of bodily awareness and reduce distress (Burrows, 2013; Payne et al., 2016; Stirling & Andrews, 2021; Yehuda, 2005).
(2) When determining what grounding techniques work for the client, pay attention to their reactions. Clients may not be aware of their triggers, so it is possible that a grounding stimulus may unknowingly trigger them (Bayne & Thompson, 2018).
(3) Expect to implement grounding techniques more frequently in the early stages of treatment; use will likely attenuate with client treatment gains (Brand et al., 2012).
(4) Practice grounding techniques with clients when they are calm and emotionally regulated (i.e., not experiencing dissociation) and discuss with clients that grounding techniques may not work immediately upon application.
(5) If a client begins to dissociate, communicate with the client in a calm, gentle manner. If the client cannot respond, begin the gradual implementation of grounding techniques and continue using the techniques until the client is stabilized. Clinicians should be thoughtful and use their clinical judgment, after consultation with the client, to determine whether or not it is appropriate to return to the material being discussed prior to the dissociative episode. Any decision should prioritize client safety.
(6) Cognitive grounding techniques may be used for cognitive reintegration and reality testing. However, if a client is verbally incoherent or unfocused, then the clinician should return to sensory grounding since cognitive processes will not function if sensory integrity is not established first (Baumard et al., 2025). Cognitive grounding techniques may function as an indirect approach to grounding.

A non-exhaustive list of common grounding techniques described within the reviewed literature is presented below (Table 4). Please note that the listed techniques have not been empirically evaluated.

**Table 4.** Commonly Referenced Grounding Techniques.

Technique	Targeted sense
Name five things that you can see.	Sight
Name five things that are a certain color.	Sight
Name four things you smell.	Olfactory
Smell something with a strong scent.	Olfactory
Run cold water over your hands.	Tactile
Touch a rock.	Tactile
Feel the chair you are sitting in and how it feels against you.	Tactile
Take a sip of water.	Taste
Sucking on a mint.	Taste
Listen to music.	Auditory
What can you hear around you?	Auditory

Compiled from Farrell and Taylor (2017), Paulik et al. (2020), Smout (2018), and Welfare-Wilson et al. (2021).

Therapy is a collaborative endeavor, and the use of grounding techniques in these settings should facilitate trust in the therapeutic relationship. One of the central principles of the American Psychological Association’s Code of Ethics (APA, 2017b) is respect for people’s rights and dignity, and these techniques help clients reestablish a sense of bodily autonomy and self-control. For many trauma survivors, grounding techniques offer the first experience of bodily safety since the trauma occurred. For this reason, grounding techniques warrant consideration as part of the treatment plan for any client at risk for dissociation.

### Limitations

Conclusions drawn from the current review must be considered within the context of some limitations. Due to the lack of standardized terminology for grounding techniques, it is possible that some potential sources were not identified for inclusion. Other interchangeable terms identified included exercises, strategies, activities, elements, and methods (Kaur et al., 2016). Future research in this area will need to expand the search parameters to these terms as well. In addition, the identification of common characteristics of therapeutic grounding in the literature was predominantly based on anecdotal reports and thus limited by methodological bias. Likewise, a majority of the sources utilized in this review (*n* = 12) only briefly mention grounding techniques. While the remaining studies (*n* = 7) presented much more information about grounding techniques, discussion of the extant descriptions and definitions of grounding in this review are limited to these sources. The proposed definition is currently only applicable to dissociative processes rather than other clinical phenomena.

## Conclusion

Grounding techniques are widely used for the treatment of disorders with dissociative characteristics, but few empirical investigations actually justify their use. In addition to the lack of a common definition for therapeutic grounding, there is no centralized terminology, which makes it very difficult to systematically and comprehensively evaluate the extant literature on the matter. When grounding techniques are described in the literature, most descriptions mention sensory input and cognitive recall. The definition proposed by this review expands on previous definitions to permit evaluation of grounding techniques via the scientific method. Specifically, the proposed definition is particularly amenable to the measurement of physiological change. Additionally, it is recommended that further research on the topic be conducted consistently to permit systematic research and necessary scientific scrutiny. Moreover, focused research on this topic may enhance the application and real-world effectiveness of therapeutic grounding for individuals with disorders characterized by dissociation.

**Table:** Critical Findings.

• “Grounding techniques” are widely used in therapy to address dissociative episodes, but there is limited evidence of their effectiveness due to the lack of a meaningful operational definition of the term.
• During dissociative episodes, individuals lose access to cognitive processes, such as cognitive recall, while sensory processes remain intact.
• Mindfulness techniques could be confused with grounding techniques and may be iatrogenic during dissociative episodes.
• There is no centralized terminology; grounding techniques, exercises, strategies, and skills are all used interchangeably with other terms.
• Within the literature, grounding techniques are primarily discussed as utilizing the five senses to reorient a person back to the present.
• Hyperarousal and hypoarousal are states of physiological disequilibrium characterized by autonomic dysregulation.
• The effectiveness of grounding techniques should be measured by changes in arousal and restoration to physiological equilibrium.

**Table:** List of Implications for Practice, Policy, and Research.

Recommendations for future research	Further research is needed to examine the following
	• Evaluate the effectiveness of the proposed definition.
	• Use “grounding techniques” to refer to therapeutic grounding in order to centralize terminology.
	• Compare the effects of grounding techniques on different groups.
	• Gather data on the efficacy of grounding techniques using the proposed definition.
	• Gather data and evaluate differentiation of grounding techniques for hypo and hyperaroused dissociation.
Implications for policy and practice	Recommendations for policy and practice include the following
	• All components used in therapy need to have a clear, measurable operational definition in order for the effectiveness of a technique to be measured.
	• Centralized terminology allows for clear communication about the utility of techniques between researchers, therapists, and the lay community.
	• Develop guidelines for the implementation of grounding techniques in therapeutic settings.
	• Gain further understanding about the physiological changes occurring during the therapeutic process.
	• Increase our understanding of grounding techniques and which techniques are most effective for use with hypo and hyperaroused individuals.
